# Plus moist HS-W^®^: a new nasal packing material for the middle meatus in endoscopic sinus surgery

**DOI:** 10.1007/s00405-023-08437-4

**Published:** 2024-01-14

**Authors:** Ryo Wakasugi, Takanobu Sasaki, Satoshi Takano, Hisashi Kamada, Kuniaki Yoshioka, Kaori Tochigi, Ryo Ikeda, Nao Takahashi, Hiroshi Matsuyama, Arata Horii

**Affiliations:** Department of Otolaryngology Head and Neck Surgery, Niigata University Graduate School of Medical and Dental Sciences, 1-757 Asahimachi-Dori, Chuo-Ku, Niigata City, 951-8510 Japan

**Keywords:** Plus moist HS-W^®^, Endoscopic sinus surgery, Calcium alginate, Moist wound healing, Nasal packing

## Abstract

**Purpose:**

Removal of the current calcium alginate packing materials to the middle meatus in endoscopic sinus surgery (ESS) is usually accompanied by discomfort or pain owing to the hard and brittle nature of these materials. Plus moist HS-W^®^ is a new calcium alginate packing material released in 2022 developed to overcome this issue by changing the uronic acid component. We aimed to compare the discomfort/pain during the removal of Plus moist HS-W^®^ with Kaltostat^®^, as well as their suitability as packing materials in ESS.

**Methods:**

Kaltostat^®^ and Plus moist HS-W^®^ were used as packing materials in 22 and 21 patients who underwent ESS in 2021 and 2022, respectively. Patients were asked to rate the pain during the packing removal 10 days after ESS using the Numerical Rating Scale (NRS). The ratio of residual packing materials, number of suctions (insertions/extractions of the suction cannula), and time required to remove packing materials were measured. Postoperative complications such as hemorrhage, local infection, lateralization of the middle turbinate, and synechia of the middle meatus were also evaluated.

**Results:**

The Plus moist HS-W^®^ group exhibited significantly lower NRS pain scores, a lower ratio of residual packing materials, a reduced number of suctions, and a shorter time required to remove the packing. No obvious postoperative complications occurred in both groups except for one suspicious case of a slight infection in the Kaltostat^®^ group.

**Conclusion:**

Compared with Kaltostat^®^, Plus moist HS-W^®^, characterized by better gelatinization than Kaltostat^®^, benefits patients by minimizing discomfort/pain during removal.

**Level of evidence:**

Level 3.

## Introduction

The ideal requirements for nasal packing materials in the middle meatus in endoscopic sinus surgery (ESS) are to regulate postoperative hemorrhage, promote faster healing of damaged mucosa, and prevent adhesions [1]. Calcium alginate exhibiting a strong hemostatic effect and facilitates moist wound healing by absorbing wound exudates and effective gelatinization [2], is one of the most frequently utilized packing materials in ESS. The current calcium alginate products such as Kaltostat^®^ (ConvaTec, UK) meet these requirements as packing materials; however, discomfort or pain during packing removal owing to its hard and brittle nature is a well-known clinical concern. Plus moist HS-W^®^ (Zuiko Medical, Japan), a new calcium alginate product released in 2022, was developed to overcome this issue by changing the uronic acid component to exhibit better gelatinization. In this study, we aimed to assess the discomfort/pain during Plus moist HS-W^®^ packing removal and its requirements as packing materials and compared it with Kaltostat^®^.

## Materials and methods

### Ethical considerations

This study was approved by the institutional review board (IRB) of our institution (IRB No. 2022-0066) and followed the principles of the Declaration of Helsinki by the World Medical Association.

### Study design

This was a prospective cohort study conducted at our institution and its associated hospitals between October 2021 and May 2022.

### Patients

Patients in this study constituted a cohort of 43 individuals who underwent bilateral ESS under general anesthesia to treat bilateral chronic rhinosinusitis (CRS). The first group, referred to as the Kaltostat^®^ group, consisted of 22 patients treated between October and December 2021. The second group, known as the Plus moist HS-W^®^ group, consisted of 21 patients treated between January and May 2022. Informed consent was obtained from all the participants prior to the study.

Diagnosis of CRS was based on medical history, nasal endoscopy, and computed tomography (CT) findings. The study’s inclusion criteria were (1) 20 years of age, (2) resistance to medical treatment necessitating bilateral ESS. The exclusion criteria comprised of (1) presence of odontogenic sinusitis or fungal sinusitis, and (2) ongoing treatment with antiplatelet drugs or anticoagulants.

### Surgical procedure and insertion of packing materials

The study participants underwent ESS at the Departments of Otolaryngology-Head and Neck Surgery at Niigata University and its associated hospitals.

Regarding the extent of ESS, frontal and sphenoid sinus was not opened in 2 of the 22 patients in the Kaltostat^®^ group. In comparison, a comprehensive approach involving the opening of all sinuses was employed in all 21 Plus moist HS-W^®^ group patients. Septoplasty was performed on 18 of 22 patients in the Kaltostat^®^ group and 12 of 21 patients in the Plus moist HS-W^®^ group, respectively.

**Fig. 1:**
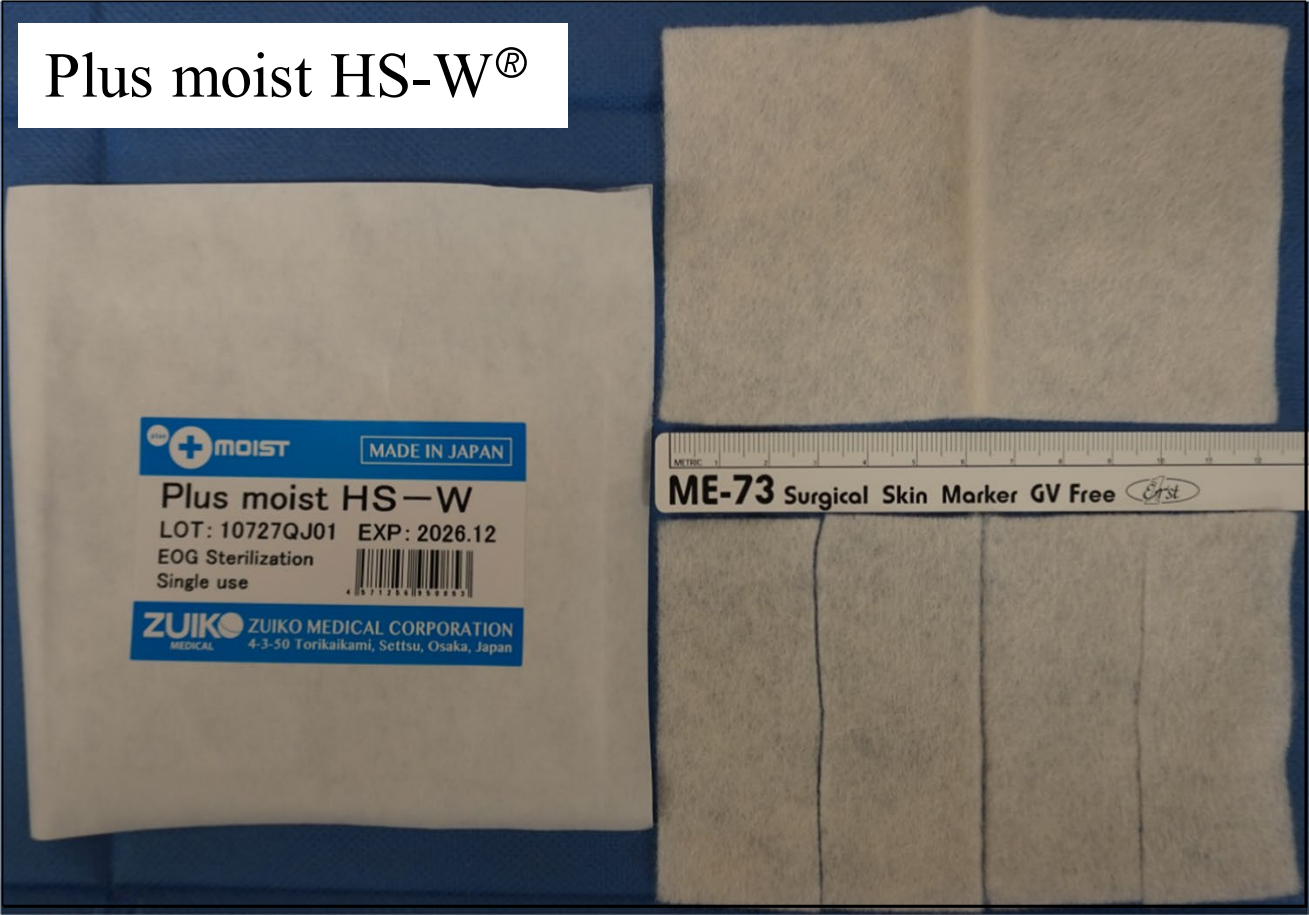
Plus moist HS-W®. Plus moist HS-W^®^, a new calcium alginate product measuring 80 × 120 mm in diameter, is cut into four pieces when being packed into the middle meatus

The Kaltostat^®^ sheets measured 75 × 120 mm, while the Plus moist HS-W^®^ sheets measured 80 × 120 mm (Fig. 1). Each sheet was cut into four pieces, with two pieces inserted into each side of the middle meatus after ESS. Following the placement of packing material in the middle meatus, a Merocel^®^ (Medtronic, USA), a polyvinyl alcohol tampon, was inserted to pack the common nasal meatus to prevent possible post-septoplasty complications including septal bleeding and hematoma and nasal synechiae [3], while the routine nasal packing after septoturbinoplasty is still controversial [4]. To control conditions and make comparisons possible between the patients, Merocel^®^ packing was also performed for those who did not receive septoplasties. In clinical setting, additional packing may not be necessary when septoplasty was not conducted.

### Postoperative care

Merocel^®^ was removed on the second or third postoperative day. Subsequently, patients were advised to perform nasal irrigation with 150–240 mL of saline 2–4 times daily and cotton ball packing placed on both sides. Packing materials that migrated from the middle meatus to the common nasal meatus were removed by suctioning.

In both groups, intravenous drip infusion of cefotiam 1 g was administered during and after the ESS and also twice daily on the first and second postoperative days. Oral administration of clarithromycin (CAM) 400 mg/day was initiated from the third postoperative day, which was later reduced to 200 mg/day. Although World Health Organization (WHO) recommends against continuing antibiotic prophylaxis postoperatively for any type of surgery for the purpose of preventing surgical site infections (Strong recommendation/moderate quality of evidence) [5], the majority of otolaryngologists report prescribing prophylactic postoperative antibiotics for patients undergoing ESS for medically refractory CRS (from 62.3% [6] to 86.8% [7]). We also administered antibiotics after ESS according to the standard procedure described in Japanese textbook [8], but we may need to reevaluate the necessity of this procedure in the future.

Analgesics were used as needed, with acetaminophen administered orally or via a suppository, adjusted according to the patient’s weight. The median period of hospitalization after ESS was 6 days ranging from 3 to 7 days.

### Outcome assessment

Approximately, 10 days after surgery, Kaltostat^®^ or Plus moist HS-W^®^ was removed by suctioning and/or using forceps. A suction cannula with an outer diameter of φ2.7–3.3 mm was used for suctioning.

Patients’ discomfort/pain score during the packing material removal was assessed using the Numerical Rating Scale (NRS) [9]. The NRS was rated using a questionnaire with a scale from 0 to 10, with 0 being the state before removal, and 10 signifying the highest pain intensity encountered by the patient. Cases in which no removal procedures were necessary (daily nasal irrigations had already washed out the packing materials completely at the time of removal) were scored as 0.

The ratio of residual packing material, number of suctions, and time required to remove the packing material were measured. Nasal residuals were assessed in increments of 10% compared with the endoscopic findings during Merocel^®^ removal; If only a small amount of fiber remained, it was categorized as 5%, and no residuals were recorded as 0%. The number of suctions indicates the number of times the suction cannula was inserted/extracted per nasal cavity to eliminate any residual packing materials.

Postoperative complications, hemorrhage, local infection, lateralization of the middle turbinate, and synechia of the middle meatus were also evaluated through endoscopic digital photography at 2–3 days, approximately 10 days, and 4 weeks after the surgery.

### Statistical methods

The nonparametric Mann–Whitney *U* and Fisher’s exact tests were used to compare demographic and clinical data between the Kaltostat^®^ and Plus moist HS-W® groups. The NRS of pain experienced during packing removal, number of suctions, and time required for packing removal were compared between the groups using the nonparametric Mann–Whitney *U* test. EZR version 1.55 was used as the statistical analysis software [10].

## Results

Table 1 presents the patient demographic data for the Kaltostat^®^ group and the Plus moist HS-W^®^ group, encompassing variables such as age, sex, asthma status, peripheral eosinophil count, eosinophil count of nasal polyps, preoperative Lund–Mackay scores, operation time, intraoperative bleeding, the day Merocel^®^ was removed, hospitalization periods after ESS, the day packing material was removed, and years of the surgeon’s otorhinolaryngology experience. No significant differences were observed among all factors between the two groups.

**Table 1:** Patient background

	Kaltostat® group	Plus moist HS-W® group	P value
Cases	22	21	
Age	58.5 [21–75]	56.0 [29–79]	0.47
Sex (male:female)	14:8	12:9	0.76
Asthma (%)	59.1	33.3	0.13
Peripheral eosinophil count (%)	5.7 [0.4–19.9]	4.5 [1.6–19.0]	0.97
Eosinophil count of nasal polyps (/HPF)	120 [0–2530]	132 [0–885]	0.41
Preoperative Lund-Mackay scores	10.0 [7–24]	10.8 [5–24]	0.93
Operation time (min)	176.5 [108–349]	211.0 [119–393]	0.38
Intraoperative bleeding (mL)	100 [0–740]	90 [0–290]	0.40
Septoplasty (cases)	18	12	0.08
The day Merocel^®^ was removed (day)	3 [2, 3]	3 [2, 3]	0.07
Hospitalization periods after ESS (day)	6 [3–7]	6 [3–7]	0.51
The day packing material was removed (day)	10 [7–12]	10 [7–11]	0.81
Years of ORL experience of the surgeon (years)	4 [3–19]	4 [2–9]	0.26

*HPF* high power field, *ORL* otorhinolaryngology

**Fig. 2:**
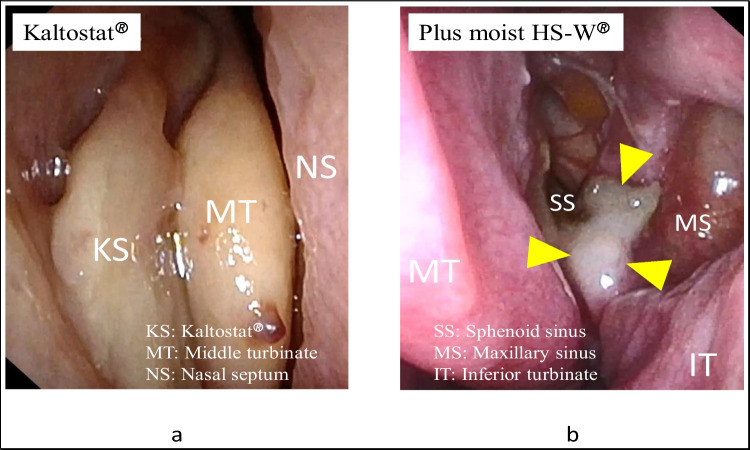
Middle meatus immediately before the removal of **a** Kaltostat^®^ and **b** Plus moist HS-W^®^ 10 days after ESS. **a** Two fragments of poorly gelatinized Kaltostat^®^ remained in the middle meatus. These fragments possessed a hard and brittle texture and easily torn off, thereby, prolonging the removal process and causing discomfort and pain to the patient. **b** A small portion of Plus moist HS-W^®^, indicated by yellow arrowheads, remained in the middle meatus but were easily removable by suction. In this case, the residual ratio was found to be 5%. *ESS* endoscopic sinus surgery, *KS* Kaltostat®, *MT* middle turbinate, *NS* nasal septum, *SS* sphenoid sinus, *MS* maxillary sinus, *IT* inferior turbinate

Figure 2 shows the typical views of the middle meatus just before the removal of packing materials. As shown in Fig. 2a, two pieces of poorly gelatinized Kaltostat^®^ remained in the middle meatus, which was hard, brittle, and easily torn off, so the removal took a longer time and was accompanied by discomfort/pain for the patient. By contrast, a small amount of Plus moist HS-W^®^, remained in the middle meatus (Fig. 2b), which was easily removed by suctioning. In this case, the residual ratio was rated as 5%.

### Packing material residues

**Fig. 3:**
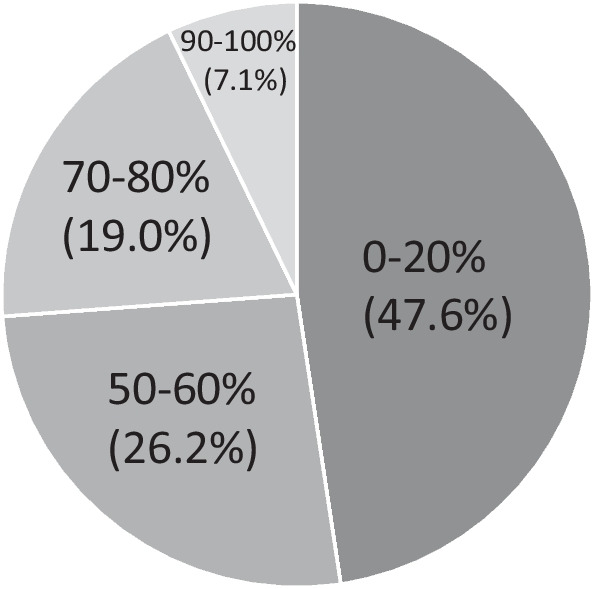
Residual ratio of Plus moist HS-W^®^. In approximately half of the patients (47.6%), only ≤ 20% of Plus moist HS-W^®^ remained. The average Plus moist HS-W^®^ residual ratio was found to be 38.0%

In 1 of the 44 nasal cavities from 22 patients of the Kaltostat group, a piece of Kaltostat^®^ had naturally fallen from the middle meatus to the common meatus at 3 days post-ESS, requiring its removal. In the other 43 cavities, there were no such instances. As a result, 87 of the 88 inserted Kaltostat^®^ pieces remained in the middle meatus without being washed out. Contrarily, Plus moist HS-W^®^ was absorbed and completely washed out (Plus moist HS-W^®^ residual ratio = 0%) in 11 of the 42 nasal cavities from 21 patients at packing removal. As shown in Fig. 3, in approximately half of the patients (47.6%), only ≤ 20% of Plus moist HS-W^®^ remained. The average Plus moist HS-W^®^ residual ratio was 38.0%.

### NRS of pain during packing removal

**Fig. 4:**
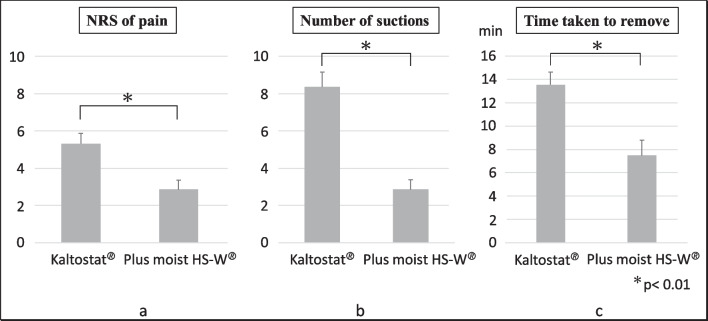
**a** NRS of pain during packing removals, **b** number of suctions during packing removals, and **c** time taken to remove packing materials. Data are mean ± SE. **a** NRS of pain scores were 5.318 ± 0.552 and 2.857 ± 0.504 in the Kaltostat^®^ and Plus moist HS-W^®^ groups, respectively, demonstrating a significant difference between the groups (*p < *0.01). **b** The number of suctions per nasal cavity was 8.364 ± 0.795 for Kaltostat^®^ and 2.857 ± 0.517 for Plus moist HS-W^®^, indicating a significant difference between the groups (*p < *0.01). **c** The time taken for removal was 13.545 ± 1.085 min for Kaltostat^®^ and 7.476 ± 1.314 min for Plus moist HS-W^®^, revealing a significant difference between the groups (*p < *0.01)

The mean ± SE NRS of pain was 5.318 ± 0.552 for the Kaltostat^®^ group, and 2.857 ± 0.504 for the Plus moist HS-W^®^ group, respectively, showing a significant difference (*p < *0.01) (Fig. 4a).

### Number of suctions required to remove packing materials

Packing removal was performed at 9.6 ± 1.4 and 9.5 ± 1.3 days after surgeries for Kaltostat^®^ and Plus moist HS-W^®^, respectively (Table 1). Figure 4b shows the number of suctions (insertions/extractions of suction cannula required to remove Kaltostat^®^ or Plus moist HS-W^®^ from the nasal cavity). The mean ± SE number of suctions per nasal cavity was 8.364 ± 0.795 for Kaltostat^®^ and 2.857 ± 0.517 for Plus moist HS-W^®^, showing a significant difference between the groups (*p < *0.01).

### Time taken to remove packing materials

Figure 4c illustrates the time required to remove Kaltostat^®^ or Plus moist HS-W^®^ from nasal cavities. The mean ± SE time taken for removal was 13.545 ± 1.085 min for Kaltostat^®^ and 7.476 ± 1.314 min for Plus moist HS-W^®^, which was significant (*p < *0.01).

### Postoperative complications

There was no postoperative hemorrhage requiring supplementary interventions and middle turbinate lateralization/middle meatus synechia in either of the groups following the surgery. There were no signs of local infection in the Plus moist HS-W^®^ group. Conversely, in one patient in the Kaltostat^®^ group, slight undeniable finding of infection in one nasal cavity was observed. The middle meatus of this patient was slightly polypoid with white fibrous remnants or a small amount of pus, which raised the possibility of infection. No patients displayed a fever of ≥ 37.5 °C in either of the groups.

## Discussion

Many studies have reported relatively good results with calcium alginate nasal packing material [2, 11, 12]. Calcium alginate has become popular owing to its low cost and the beneficial properties required for packing materials including, biocompatibility, non-toxicity, wet dressing, and high absorption and hemostat capacity [13–15]. The currently available calcium alginate packing material, such as Kaltostat^®^, is hard and brittle in nature causing discomfort or even pain to the patient during its removal. It is known that alginate’s gelatinizing properties depend on the uronic acid component [16, 17], which is crucial for ease of manipulation during packing removal. Plus moist HS-W^®^, a new calcium alginate packing material, was developed to have better gelatinizing properties by modifying the uronic acid component (from guluronic acid-rich to mannuronic acid-rich components).

In our study, only ≤ 20% of Plus moist HS-W^®^ remained at packing removal in approximately half of the patients (47.6%). The average Plus moist HS-W^®^ residual ratio was 38.0%. By contrast, except for 1 of the 88 pieces that naturally fell to the common meatus, Kaltostat^®^ remained in the middle meatus with poor gelatinization despite the daily nasal irrigation with saline. These differences may be owing to the better gelatinizing characteristics of Plus moist HS-W^®^ than those of Kaltostat^®^.

Plus moist HS-W^®^ could be removed by fewer suctions and time, perhaps partly owing to the low residual ratio. Kaltostat^®^ is manufactured from a guluronic acid-rich alginate [18], which is hard, brittle, and torn easily during removals. Unlike Plus moist HS-W^®^, which could be eliminated by only suctioning, Kaltostat^®^ required a longer time to be removed owing to forceps use and frequent suctioning (insertions/extractions of the suction cannula).

The NRS for pain during packing removals was significantly lower in the Plus moist HS-W^®^ group than in the Kaltostat^®^ group. Frequent insertions/extractions of the suction cannula, use of forceps, and the longer time required for packing removals could all affect the high NRS of pain in the Kaltostat^®^ group.

Regarding the postoperative complications, hemorrhage requiring supplementary interventions and middle turbinate lateralization/middle meatus synechia were not observed in either of the groups, suggesting that both packing materials have sufficient properties for hemostasis, wound healing, and adhesion prevention. Only 1 of the 22 patients in the Kaltostat^®^ group demonstrated a slightly suspicious sign of local infection; however, its clinical significance may be low. Overall, both Plus moist HS-W^®^ and Kaltostat^®^ meet the requirements as packing materials. These results are derived from common characteristics of both packing materials. In calcium alginate, the calcium ions act as blood coagulation factor IV to promote hemostasis [19], alginate absorbs excess exudate at the surgical wound and is then gelatinized [20], and the resultant gel creates a moist environment that promotes cellular regeneration [21] and accelerates wound healing to prevent adhesion [22].

## Conclusion

Plus moist HS-W^®^ is a new ideal calcium alginate material that meets the requirements of nasal packing materials (regulating postoperative hemorrhage, promoting moist wound healing, and preventing adhesions). Moreover, discomfort/pain during packing removals was improved with Plus moist HS-W^®^ by changing its component to achieve better gelatinization properties than those of Kaltostat^®^.

## Data Availability

The data that support the findings of this study are available from the corresponding author, [Ryo wakasugi], upon reasonable request.
